# Emphysematous pyelonephritis diagnosed by acute changes detected via computed tomography: A case report

**DOI:** 10.1002/ccr3.9211

**Published:** 2024-08-12

**Authors:** Yoichiro Kato, Shuhei Ishii, Yuta Goto, Yasushi Nozaki, Tatsuya Kawamura, Gota Morino, Shintaro Hoshi, Gaku Takahashi, Wataru Obara

**Affiliations:** ^1^ Department of Urology Iwate Medical University Iwate Japan; ^2^ Department of Critical Care, Disaster and General Medicine, School of Medicine Iwate Medical University Iwate Japan

**Keywords:** computed tomography, diabetes mellitus, emphysematous pyelonephritis, sodium‐glucose co‐transporter 2 inhibitors

## Abstract

This is the first case report in which computed tomography (CT) images of a patient with emphysematous pyelonephritis (EPN) capture the time course of emphysema from onset to resolution through conservative treatment. To re‐evaluate EPN, including refractory urinary tract infections, CT scans after 72 h might be helpful.

## 1 INTRODUCTION

Emphysematous pyelonephritis (EPN) is a type of acute pyelonephritis in which gas accumulates in and around the renal parenchyma and can be fatal. At the time of initial presentation, multidisciplinary treatment, including nephrectomy, should be introduced immediately. Although treatment algorithms for EPN have been reported in case series, the diagnostic and treatment criteria are categorized based on computed tomography (CT) image results.^1^ However, there are few reports on the time it takes from initiating treatment to observing a change in CT scan results that signify EPN resolution. Here, we report a case in which we tracked changes in CT scan results over time using multiple CT scans to monitor the EPN status of the patient during treatment.

## 2 CASE HISTORY/EXAMINATION

A 49‐year‐old woman had been receiving treatment for diabetes mellitus (DM), dysuria due to neurological bladder, adrenal insufficiency, and somatoform disorders. Based on this background, the patient was prescribed sitagliptin, dapagliflozin, nateglinide, insulin glargine for diabetes, and urapidil and distigmine bromide for dysuria. Moreover, the patient was prescribed prednisolone for adrenal insufficiency and diazepam, clonazepam, mirtazapine, trazodone, and sodium valproate for the somatoform disorder. Vomiting frequently occurred on the day before the consultation. On the morning of the consultation, her level of consciousness decreased, and the primary care physician examined her and observed a reduction in oxygen concentration of 60%–70%. The patient was immediately transferred to our hospital via emergency transportation. Immediately after arriving at the hospital, her physical evaluations revealed a body temperature of 33.3°C, blood pressure of 107/40 mmHg (under conditions where noradrenaline [NA] treatment is carried out at 0.29γ), heart rate of 127 bpm, Glasgow Coma Scale (GCS) of 10 points, E3 V3 M4, sequential organ failure assessment (SOFA) score of 15 points, and an acute physiology and chronic health evaluation II (APACHE2) score of 32 points. Biological assessment revealed altered renal function with a creatinine level of 40.7 mg/L and creatine kinase (CK) concentration of 7522 IU/L. Infection‐related parameters showed C‐reactive protein (CRP) at 321.5 mg/L, white blood cells (WBC) at 11,500/μL, hemoglobin at 12.9 g/dL, lactate at 11.6 mmol/L, and glycosylated hemoglobin A1c (HbA1c) at 9.4%.

## 3 METHODS

**FIGURE 1:**
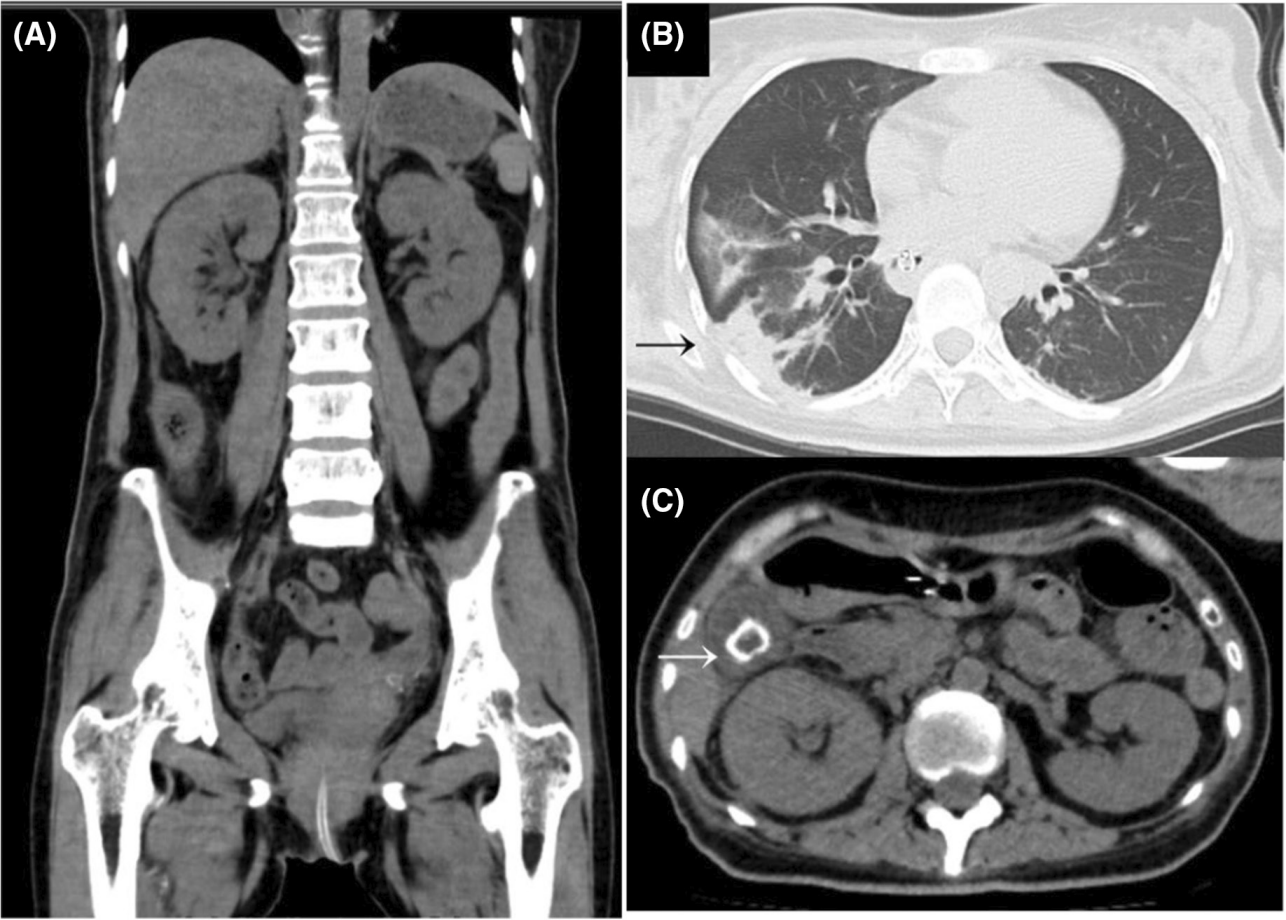
Computed tomography radiographs of a patient after arriving at the hospital. There are no obvious abnormalities in the urinary tract (A–C) other than moderate pneumonia (black arrow) (B) and gallstones (white arrow) (C).

**FIGURE 2:**
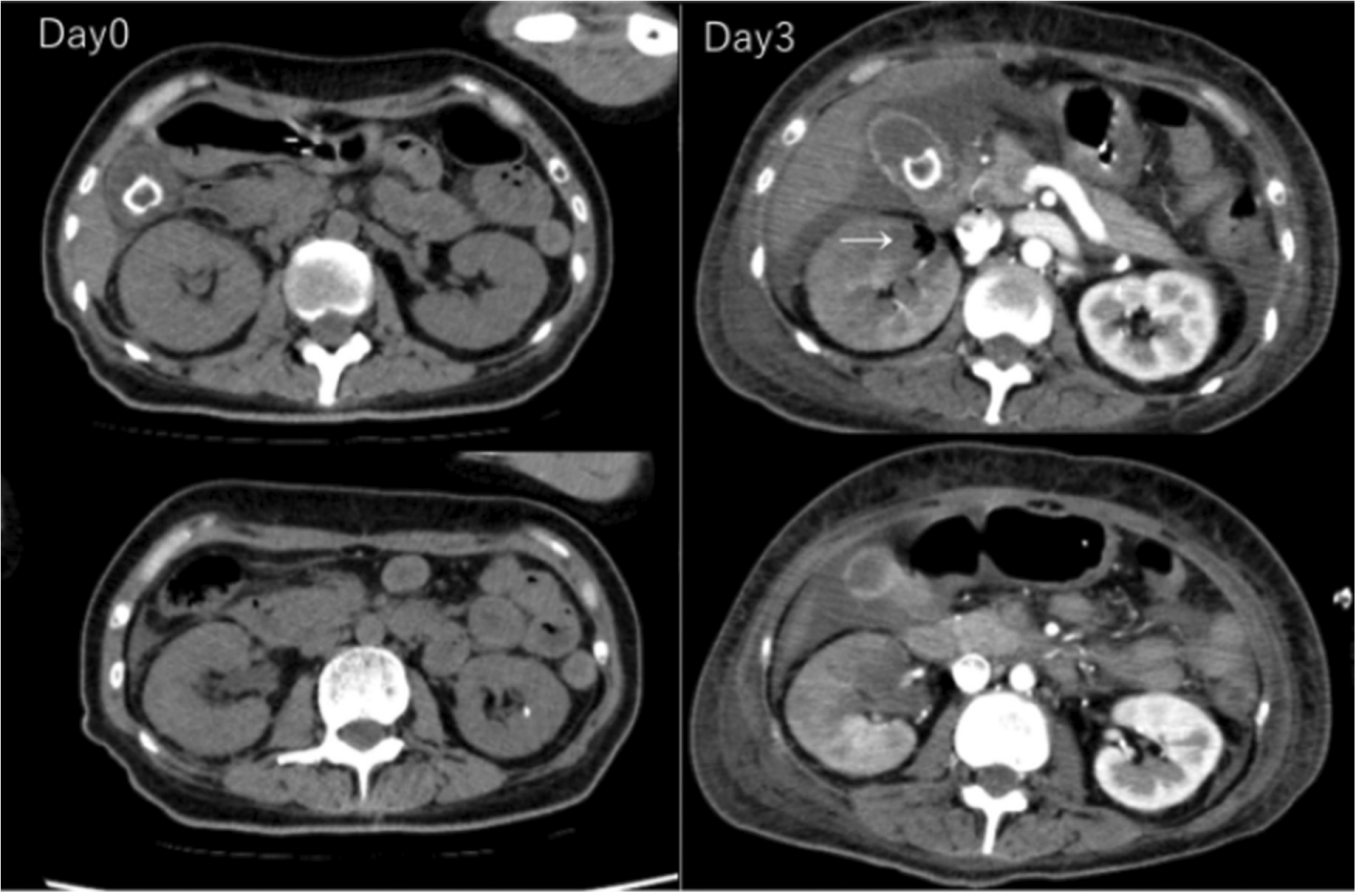
Computed tomography (CT) imaging results on the 0th and 3rd day of hospitalization. On the 3rd day of hospitalization, new renal emphysema formation was observed in the right kidney (white arrow).

In parallel with the physical examination, sputum, urine, and blood sample‐derived microbial cultures, respiration management, and central venous access were established. After analyzing the results of the microbial culture tests, a treatment regimen consisting of tazobactam/piperacillin, continuous hemodiafiltration (CHDF), polymyxin B‐immobilized fiber column direct hemoperfusion (PMX‐DHP), and regular human insulin administration was initiated. A CT scan performed after artificial respiration failed to identify any obvious infection foci other than mild pneumonia, and the patient was urgently admitted to the hospital with an infection of unknown origin (Figure 1). As no significant deterioration of vital signs was observed on the 3rd day of hospitalization, contrast‐enhanced CT was performed to detect the lesion. The results of the CT scan revealed emphysematous changes in the kidney (Figure 2). On the same day, blood microbial culture tests revealed ampicillin/ABPC‐, piperacillin/PIPC‐, and fosfomycin/FOM‐resistant *Escherichia coli*. This finding was consistent with the urine culture results. Therefore, tazobactam/piperacillin (TAZ/PIPC) was replaced with meropenem (MEPM) on the 3rd day of hospitalization. In addition, resident bacteria were detected in sputum aspiration and stool cultures. Based on these new emphysematous changes identified in the patient, the main infection was determined to be in the kidney.

**FIGURE 3:**
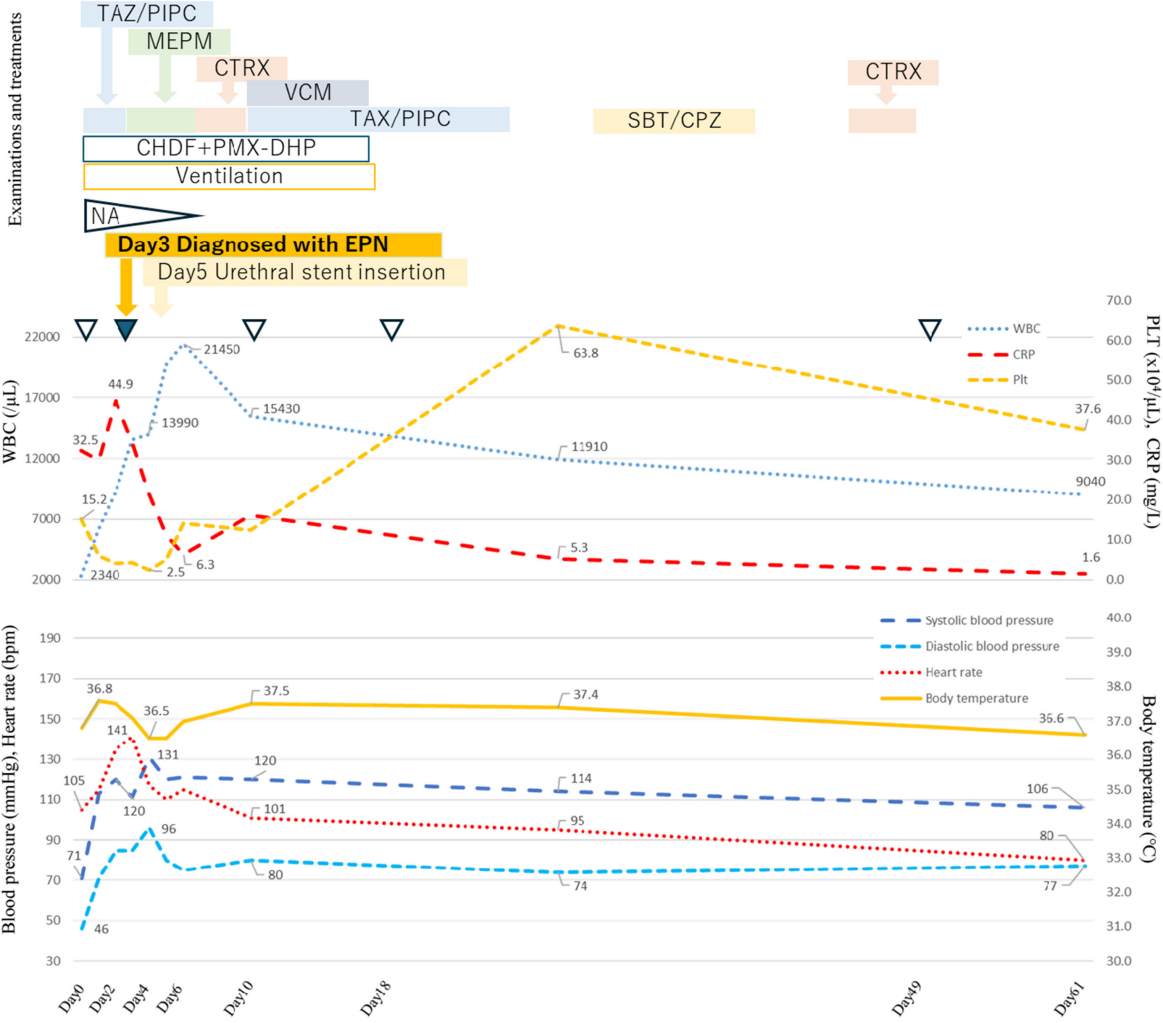
Clinical course of the current case. TAZ/PIPC tazobactam/piperacillin, MEPM meropenem, CTRX ceftriaxone, VCM vancomycin, SBT/CPZ sulbactam/cefoperazone, CHDF continuous hemodiafiltration, PMX‐DHP polymyxin B‐immobilized fiber column direct hemoperfusion, NA noradrenaline, computed tomography (white arrowhead), enhanced computed tomography (blue arrowhead), white blood cells, PLT platelets, CRP C‐reactive protein.

**FIGURE 4:**
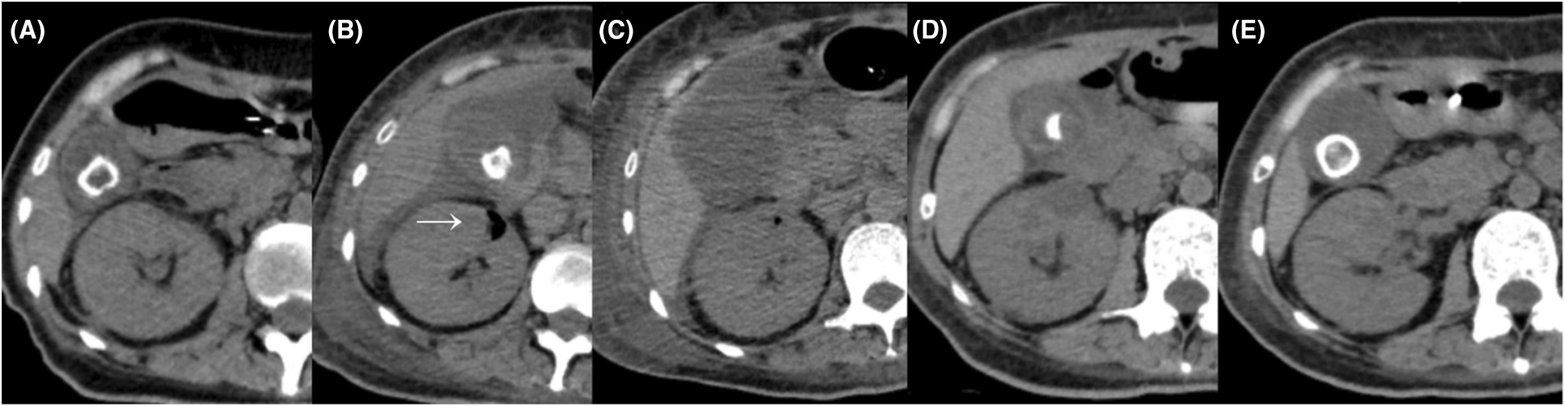
Progressive changes observed in computed tomography (CT) scan results over 49 days of hospitalization. Progressive changes were identified in CT radiographs on Day 0 (A), Day 3 (B), Day 10 (C), Day 18 (D), and Day 49 (E). The intrarenal gas detected on the 3rd day (B) (white arrow) disappeared on the 18th day (D).

Furthermore, as the emphysema was too small for puncture drainage, a ureteral stent was placed on the 5th day of hospitalization to decompress the urinary tract and secure an outflow tract. After ureteral stent placement, an increase in blood pressure and platelet count made it possible to reduce the NA dose, and the patient's level of consciousness gradually improved. On the 7th day of hospitalization, the antibiotic was changed from MEPM to ceftriaxone (CTRX) based on the culture results of de‐escalation. Furthermore, on the 10th day of hospitalization, a catheter‐related bloodstream infection (CRBSI) was suspected, and CTRX was changed to vancomycin (VCM) + TAZ/PIPC. Ventilation with a tracheal tube and administration of VCM on the 17th day of hospitalization, CHDF on the 18th day of hospitalization, and TAZ/PIPC on the 24th day of hospitalization were discontinued. Sulbactam/cefoperazone (SBT/CPX) was administered for 10 days starting on the 28th day of hospitalization, owing to the suspicion of cholecystitis. After that, CTRX was performed for a urinary tract infection associated with urethral balloon obstruction for 5 days from the 44th day of hospitalization. Figure 3 shows the treatment, procedures, and clinical course performed immediately after admission. Subsequently, follow‐up CT scans were performed on the 10th, 18th, and 49th day of hospitalization; however, no disease recurrence was observed (Figure 4). Antibiotics were discontinued on the 49th day of hospitalization, and the patient was discharged from the hospital on the 61st day. After discharge from the hospital, the ureteral stent was removed at the outpatient clinic, and the patient has not experienced any significant infection recurrence to date.

## 4 CONCLUSION

Herein, we report a case of EPN that was diagnosed using multiple CT scans throughout a 60‐day hospitalization period and was validated by systemic inflammation findings. Our findings suggest that multiple CT images showing worsening renal status may provide the information needed to enhance the current treatment regimen.

## 5 DISCUSSION

The EPN mortality rate remains extremely high (13%).^2^ The comorbidities associated with EPN include DM and urinary tract abnormalities. Among these, DM is observed in 80%–96% of patients with EPN.^1^, ^3^, ^4^ The patient described in this report also had poorly controlled DM and was taking dapagliflozin as a sodium‐glucose co‐transporter 2 inhibitors (SGLT2i).

Excretion of excess sugar into the urinary tract due to SGLT2i intake raised concerns about the risk of urinary tract infections (UTIs); therefore, this drug was discontinued after hospitalization, while DM was controlled via insulin therapy. An increasing number of studies have recently reported the absence of a negative relationship between SGLT2i and UTIs, but there is room for further debate on this question in the future.^5^, ^6^ Echeverria et al. state that a specific relationship between SGLT2 inhibitors and EPN has not been strongly established.^7^ While Nishikawara et al. suggested that urinary dysfunction might be associated with DM treatment using SGLT2i and the risk of UTI.^8^ This case had originally been prescribed urapidil and distigmine bromide for neurogenic bladder, and the results supported Nishikawara's opinion that the patient had urinary dysfunction as a background. In particular, reports of EPN associated with SGLT2i include the following: In 2018, Gupta et al. reported the first case of EPN with an SGLT2i agent for DM treatment. Between 2018 and 2023, there were four case reports of EPN while taking SGLT2i agents.^8^, ^9^, ^10^, ^11^ The causative bacteria were two cases of *E Coli* and two cases of *Candida glabrata*, respectively; therefore, this case would be the fifth case report.^8^, ^9^, ^10^, ^11^ In addition, because SGLT2i is not only for DM but also used for heart failure and chronic kidney disease, following up on cases in which it is used for purposes other than DM treatment to see whether EPN occurs might strengthen the relationship between SGLT2i and EPN.^12^ In the case of our patient, the SOFA score at the time of admission to the Intensive Care Unit was 12 points or higher and had increased to its worst value within 48 h. The mortality rate has been reported to be 50% or higher.^13^ Furthermore, although this is old data, the in‐hospital mortality rate was estimated to be 71% based on APACHE2 Scores.^14^ There are several risk classifications for EPN based on CT findings. Still, Huang et al. have classified EPN cases into four categories based on the location of gas and abscess accumulation.

Class 1: gas only in the collecting system (called emphysematous pyelitis).

Class 2: gas in the renal parenchyma without extension to the extrarenal space.

Class 3A: extension of the gas or abscess to the perinephric space.

Class 3B: extension of the gas or abscess to the pararenal space.

Class 4: bilateral EPN or solitary kidney with EPN. Based on this classification, the patient described herein was classified as a Class 2 case of EPN. Moreover, Huang et al. suggested treatment methods for each class, and for Classes 1 and 2, they recommended blood sugar control, antibiotic administration, and percutaneous drainage. Because the emphysema was small, ureteral stent placement was selected.^1^ As a result, the treatment was effective, and we avoided nephrectomy. Pontin et al. commented that signs of ureteral obstruction should be managed with percutaneous nephrostomy or ureteral stenting unless nephrectomy is necessary. However, in cases of urinary dysfunction that are thought to be caused by diabetes, as in this case, we believe that ureteral stent placement might be acceptable as a functional urinary passage disorder even in the absence of structural ureteral obstruction.^15^ Our present findings make it clear that EPN causes dramatic changes in the CT scan radiographs of the kidneys over a short period. There have been no reports on the time course of emphysema from onset to resolution in the CT images of patients with EPN. Generally, for urinary tract infections that have a poor response to initial treatment, it is recommended to re‐examine CT after 72 h to rule out renal and perinephric abscess.^8^, ^16^, ^17^ Among them, Nishikawara reported a case in which a fever continued for 72 h after the start of pyelonephritis treatment, and an additional CT scan on the 4th day led to the diagnosis of EPN. They comment on the usefulness of additional CT for the case.^8^ Although a diagnosis of urinary tract infection was not made in our case at the time of admission, a contrast‐enhanced CT scan was performed on the third day to search for infection foci, and a diagnosis of EPN was made. From this point of view, additional CT examinations are helpful for cases of infection that have lasted for more than 72 h and are difficult to treat when diagnosing EPN. Because the CRP had improved from 16.2 to 5.3 mg/L on the 18th day, the follow‐up CT was performed at this point, which revealed that the emphysema formation was disappearance. As for the disappearance of emphysema, Misgar et al. reported two cases in their case series in which emphysema disappeared during the fourth week of treatment.^18^ These two cases were Class 3B and Class 4, and the severity level on the images was higher than in this case. Furthermore, invasive procedures are unknown. For this reason, although this is just one case experience, the appropriate timing for follow‐up CT in Class 2 EPN on the condition that the patient has responded positively to treatment and CRP is improved might be around the 18th day. Our results suggest that more than multidisciplinary treatment administered during the first 4 days of hospitalization would be required. Moreover, it was suggested that multiple CT scans conducted over time can be a valuable diagnostic tool not only for detecting the location of the main lesion but also for re‐evaluation and modification of the initial treatment regimen.

## AUTHOR CONTRIBUTIONS

**Yoichiro Kato:** Conceptualization; writing – original draft. **Shuhei Ishii:** Data curation; investigation. **Yuta Goto:** Data curation; investigation. **Yasushi Nozaki:** Data curation; investigation. **Tatsuya Kawamura:** Data curation; investigation. **Gota Morino:** Investigation; resources. **Shintaro Hoshi:** Data curation; resources. **Gaku Takahashi:** Data curation; resources; supervision. **Wataru Obara:** Conceptualization; project administration; supervision; writing – review and editing.

## FUNDING INFORMATION

No funding.

## CONFLICT OF INTEREST STATEMENT

The authors declare no conflict of interest.

## CONSENT

Written informed consent was obtained from the patient for the publication of this report in accordance with the journal's patient consent policy.

## Data Availability

Data sharing is not applicable to this article as no new data were created or analyzed in this study.
